# The impact of non-severe burn injury on cardiac function and long-term cardiovascular pathology

**DOI:** 10.1038/srep34650

**Published:** 2016-10-03

**Authors:** Emily O’Halloran, Amit Shah, Lawrence Dembo, Livia Hool, Helena Viola, Christine Grey, James Boyd, Tomas O’Neill, Fiona Wood, Janine Duke, Mark Fear

**Affiliations:** 1 Burn injury research unit, University of Western Australia, Crawley, WA, Australia; 2 Cardiology unit, Fiona Stanley Hospital, Murdoch, WA, Australia; 3 School of Anatomy, Physiology and Human Biology, University of Western Australia, Crawley, WA, Australia; 4 Victor Chang Cardiac Research Institute, Sydney, NSW, Australia; 5 Centre for Data Linkage, Curtin University, Western Australia, Perth, Australia; 6 Burns Service of Western Australia, Fiona Stanley and Princess Margaret Hospital, Western Australia, Perth, Australia

## Abstract

Severe burn injury significantly affects cardiovascular function for up to 3 years. However, whether this leads to long-term pathology is unknown. The impact of non-severe burn injury, which accounts for over 80% of admissions in developed countries, has not been investigated. Using a rodent model of non-severe burn injury with subsequent echocardiography we showed significantly increased left ventricular end systolic diameter (LVESD) and ventricular wall thickness at up to 3 months post-injury. Use of propranolol abrogated the changes in cardiac measures observed. Subsequently we investigated changes in a patient cohort with non-severe injury. Echocardiography measured at baseline and at 3 months post-injury showed increased LVESD at 3 months and significantly decreased posterior wall diameter. Finally, 32 years of Western Australian hospital records were used to investigate the incidence of cardiovascular disease admissions after burn injury. People who had experienced a burn had increased hospital admissions and length of stay for cardiovascular diseases when compared to a matched uninjured cohort. This study presents animal, patient and population data that strongly suggest non-severe burn injury has significant effects on cardiovascular function and long-term morbidity in some burn patients. Identification of patients at risk will promote better intervention and outcomes for burn patients.

In the acute phase after burn injury increased sympathetic activity is a critical part of the coordinated response, important for the modulation of energy substrate mobilization, cardiovascular and hemodynamic compensation and wound repair^1^,^2^. However, prolonged or excessive sympathetic activity can also be deleterious. The hypermetabolic response to severe burn injury, largely mediated by catecholamines and corticosteroids, leads to insulin resistance/hyperglycaemia, increased infection risk and muscle protein degradation and disruption to multiple body systems^3^,^4^. Prolonged hypermetabolic changes have been shown to persist for up to 3 years post severe burn injury^5^.

Propranolol, a non-selective β-adrenergic receptor antagonist, mitigates the actions of plasma catecholamines and significantly reduces the hyperdynamic and hypermetabolic state in patients with acute burn injury. Administration of this drug for 2 weeks to decrease admission heart rate by 15% augments net protein balance in muscle, decreasing loss of lean mass and lowering resting energy expenditure^6^. Clinical trials of propranolol in both children and adults with severe burn injury are ongoing, with current reported findings that the use of propranolol is both safe and beneficial^7^,^8^,^9^.

Despite what is known about the impact of severe burn injury on catecholamines and cardiac function^4^,^5^, little work to date has studied the impact of non-severe burn injury and there have been few publications on possible long-term effects. This is despite the fact that in developed countries the vast majority of all burn injuries and even hospital presentations for burn injury are for non-severe injuries (<10% total body surface area (TBSA))^10^,^11^,^12^. Therefore it is important to determine if these smaller injuries have a significant long-term impact and if burn injury can lead to cardiac pathology. Here, we have investigated the impact of non-severe burn injury on cardiovascular function using an animal model, patient study and a population-based study using linked health administrative data.

## Results

### Non-severe burn injury leads to significant changes in cardiac parameters measured using echocardiography in mice

**Figure 1:**
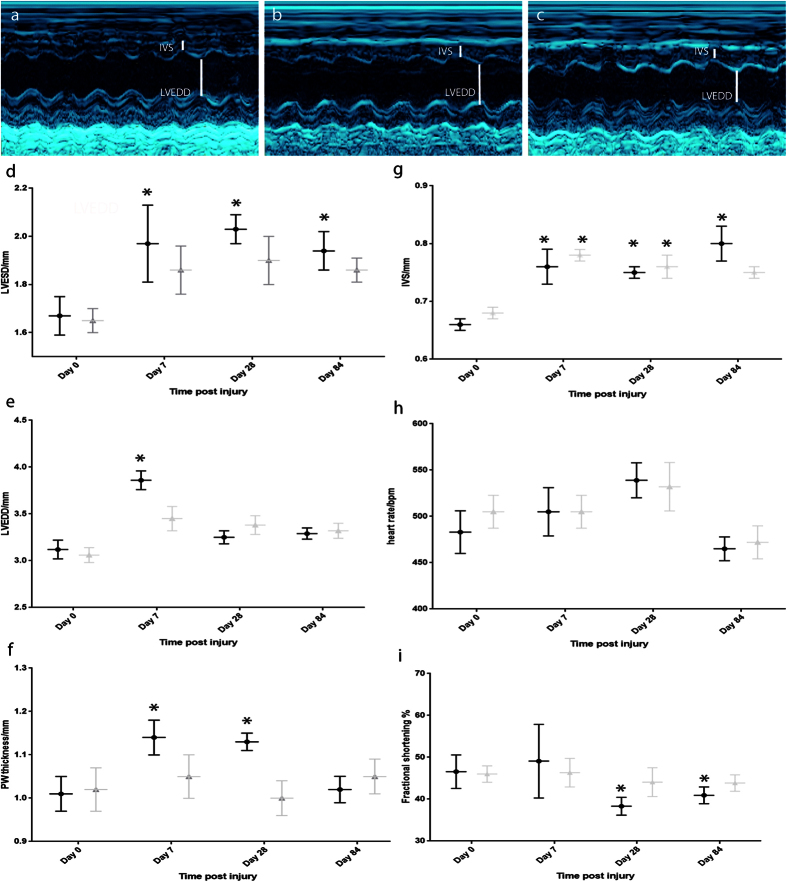
Impact of non-severe burn injury on cardiac function in mice and effect of propranolol administration. Echocardiography was conducted on mice at baseline, 1 week, 4 weeks and 12 weeks post-injury using an i13L probe on a Vivid 7 Dimension cardiac ultrasound system with rodent software (GE Healthcare). Representative images of baseline (n = 16), 12 weeks post-injury untreated (n = 8) and 12 week post injury treated with propranolol (n = 8) respectively are shown (**a**–**c**). LVESD is significantly elevated at all time-points post-injury in the control group (**d**). Use of propranolol ameliorates this change (**d**). LVEDD (**e**) and Posterior wall thickness (**f**) are both transiently elevated in control group but not in propranolol treated group (**e**,**f**). IVS thickness is elevated up to 3 months post injury in control but only up to 28 days post-injury in propranolol treated group (**g**). No significant difference is observed in heart rate in either group (**h**). Fractional shortening is significantly decreased in control at 28 and 84 days post-injury (**i**). Black circles indicate control group. Grey triangles propranolol treated group. *Indicates significantly different (p < 0.05) when compared to baseline pre-injury values.

Mice received a non-severe full-thickness burn injury (approx. 8% total body surface area) and echocardiography was conducted on mice at baseline (prior to injury), 1 week, 4 weeks and 12 weeks post-injury (n = 8 mice, Fig. 1a–c). Left ventricular end systolic diameter (LVESD) was significantly increased post burn injury by day 7 when compared to the baseline day 0 (pre-injury) measures (Fig. 1d). This increase was sustained through day 28 and up to 3 months post-injury, the final time-point tested (Fig. 1d). Left ventricular end diastolic diameter (LVEDD) also increased at 7 days post-injury (Fig. 1e), although no significant increase was sustained beyond this time-point. In addition to the increased ventricular diameter in systole, there was an increase in the posterior wall (PW) and intraventricular septum (IVS) thickness, both observed by day 7 post-injury and sustained through to 28 days post-injury, with the increased thickness of the IVS sustained to 3-months post-injury (Fig. 1f,g). No significant change in heart rate was observed when compared to baseline values (pre-injury, day 0, Fig. 1h). Fractional shortening was significantly decreased at day 28 and 84 post-injury in the control group (Fig. 1i).

### Propranolol treatment ameliorates changes in cardiac parameters observed after non-severe burn injury in mice

An additional group of mice received propranolol in drinking water after the same burn injury (n = 8) and were monitored using echocardiography at baseline, 1 week, 4 weeks and 12 weeks post-injury. The significant changes in ventricular diameter and in PW thickness were ameliorated by the administration of propranolol (Fig. 1c,d-fd) such that these measures were no longer significantly different to baseline values. The IVS was significantly thicker even in the propranolol treated group at up to 28 days post-injury, but this was not sustained to the 3 month time-point (Fig. 1g). Fractional shortening was not significantly decreased in the propranolol treated group at either day 28 or day 84, suggesting propranolol had ameliorated the changes in left ventricular contraction observed in the control group. By 3 months post-injury, no significant changes in any cardiac parameters were observed in the group treated with propranolol, suggesting propranolol treatment ameliorated the sustained changes observed in the untreated control group.

### Cardiac changes are observed at 3 months post-injury compared to baseline measures in patients with non-severe burn injury

Thirty patients were recruited for an observational study to determine whether the changes in cardiac parameters observed in the mouse study could also be seen in patients with non-severe burn. Of the thirty patients recruited to the study 24 completed the echocardiography at both baseline and 3 months post-injury. Of those completing the study, 3 were female and 21 male, reflecting the gender imbalance in the burn patient population. Age of participants ranged from 19–70 years with a median age of 27. Total body surface area of the injury ranged from 1% TBSA to 15% TBSA with a median TBSA of 4.5% (Table 1).

**Figure 2:**
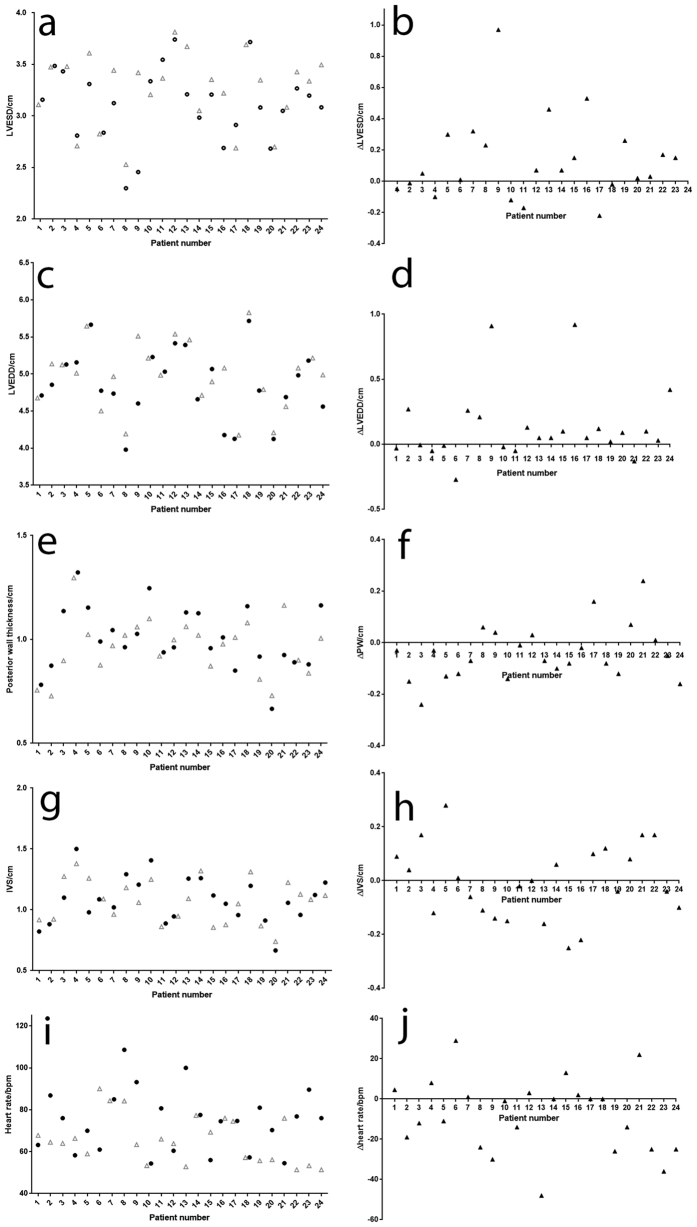
Changes in cardiac parameters after non-severe burn injury in adult patients. LVESD is significantly elevated at 3-months post-injury in the patient cohort (**a**,**b**). Posterior wall thickness is also significantly different at 3-months post injury but is reduced when compared to baseline (**e**,**f**). No other significant changes are observed in LVEDD (**c**,**d**), IVS thickness (**g**,**h**) or heart rate (**i**,**j**). Panels a, c, e, g, i show baseline measures (black circles) and 3-month post-injury measures (grey triangles) for LVESD, LVEDD, PW thickness, IVS thickness and heart rate for each patient. Panels b, d, f, h and j show the difference between 3-month and baseline measures (3-month value-baseline value) for LVESD, LVEDD, PW thickness, IVS thickness and heart rate respectively.

Similar to the findings in the animal model, patients were observed to have a significant increase in left ventricular diameter in systole at 3 months post-injury when compared to baseline (p = 0.015, Fig. 2a,b). No significant difference in diastolic diameter or heart rate was observed (Fig. 2c,d,i,j). In contrast to the animal model findings, the posterior wall thickness was significantly different at 3 month post-injury (Fig. 2e,f, p = 0.0375) but this was due to a decrease in wall thickness at 3 months when compared to the baseline rather than an increase. IVS thickness was not significantly different between baseline and 3-months post-injury in the patients (Fig. 2g,h). Fractional shortening was not significantly reduced by 3 months post-injury when compared to baseline values (data not shown) with no patient showing indications of changes at a level considered to have clinical impact. No interventional trial was conducted in the patients with non-severe injury and so whether propranolol would have the same effect as observed in mice on this group of patients is not clear.

### Population-based study cohort characteristics

The study included data of 14,555 persons aged 15 to 45 years with a first burn injury admission and 56,822 persons in a non-injury control cohort matched on age and gender. The burn cohort had an average follow up time of 16.7 years for a total of 241,366 person years (PY) of observation. The uninjured cohort had an average follow-up time of 17.0 years for a total of 965,766 PYs of observation. The median (interquartile range range) age at index for both cohorts was 27 (21–34) years and 75% of each cohort were male. Refer to Table 2 for cohort characteristics. In the burn cohort, 7% had severe burns (≥TBSA 20%), 16% full thickness burns, 37% partial thickness, 20% erythema and 29% unspecified burn depth; some patients had multiple burn sites and depths recorded.

### The burn injury cohort had an elevated risk of hospital admissions and longer length of stay for cardiovascular diseases compared to a matched non-injured control cohort

**Figure 3:**
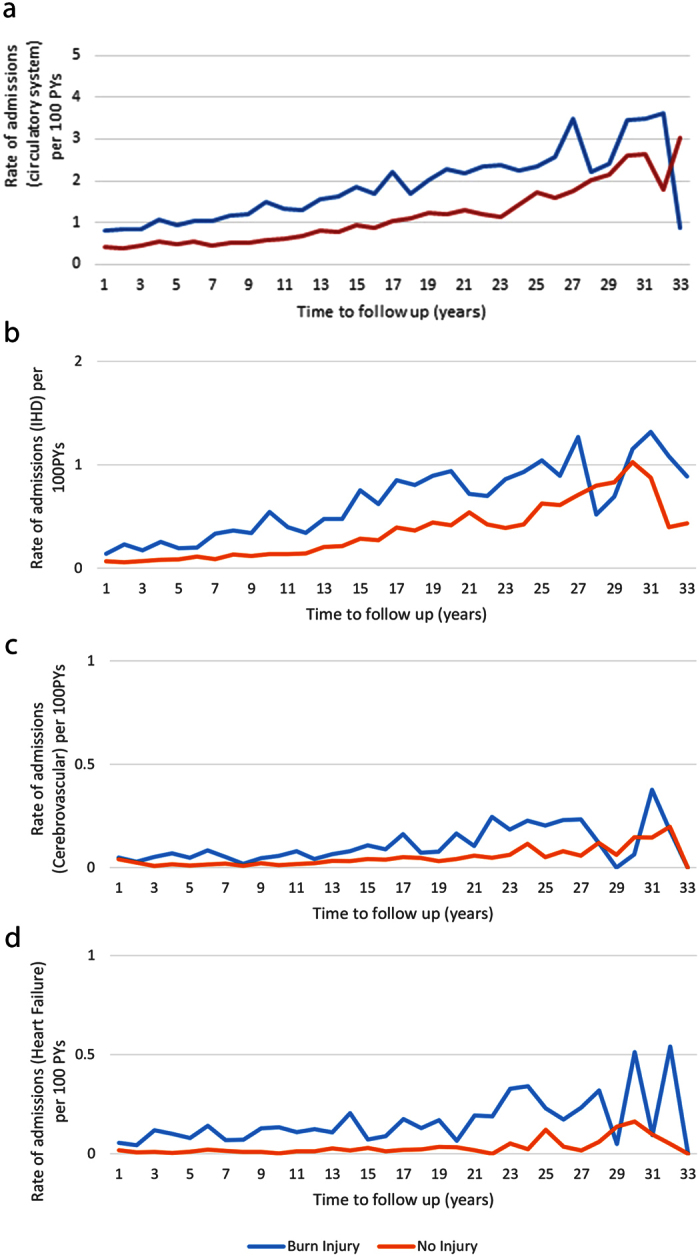
Hospital admission rates for diseases of the circulatory system. Unadjusted rates of hospital admissions (per 100 person years (PYs)) for diseases of the circulatory system (total (**a**)) and for ischaemic heart disease (**b**) (IHD), cerebrovascular disease (**c**) and heart failure (**d**) among those aged 15–44 years with burn injury versus no injury.

There were 3,613 hospital admissions with a primary diagnosis of a circulatory system disease occurring after burn injury discharge. Ischeamic heart disease was the most common cardiovascular disease sub-category accounting for almost a third of all circulatory admissions (Table 3). Unadjusted annual incidence rates for circulatory system admissions (combined) (Fig. 3a) and for the sub conditions, ischaemic heart disease (Fig. 3b), cerebrovascular disease (Fig. 3c) and heart failure (Fig. 3d), showed consistently higher rates of admissions in the burn cohort over the 33 year study compared with the uninjured cohort.

After adjusting for demographic factors and pre-existing health status, those hospitalised for a burn injury experienced 1.56 (95% CI: 1.42–1.71) times as many circulatory system disease admissions, and 2.62 times the number of days in hospital with a circulatory system diagnosis (95% CI: 1.73-3.96) than the uninjured control group. After controlling for known confounders, the burn cohort had significantly higher rates of admissions for ischaemic heart disease (IRR, 95% CI: 1.68, 1.43–1.97), cerebrovascular disease (IRR, 95% CI: 1.64, 1.26–2.13) and heart failure (IRR, 95% CI: 3.09, 1.72–5.56), compared to the uninjured cohort. Members of the burn cohort also spent longer in hospital for ischaemic heart disease (IRR, 95% CI: 2.03, 1.49–2.76), cerebrovascular disease (IRR, 95% CI: 4.34, 1.75–10.73) and heart failure (IRR, 95% CI: 4.18, 1.82–9.61), when compared with the non-injured cohort (data not shown). Subgroup analysis by cardiovascular disease and by burn severity found elevated admission rates for severe and minor burns; however, these results did not reach significance (results not presented).

### Patients with no previous admission for cardiovascular disease also showed elevated risk of admission compared to the non-injured cohort

Survival analyses were performed on data excluding those with a prior history of circulatory disease hospitalisation, and additionally, excluding those in the burn cohort with a record of a principal diagnosis injury admission(s). The time until first (incident) post-burn admissions for ischaemic heart disease, cerebrovascular disease, and first heart failure were used as event measures. Survival analysis was performed on 2,951 patients with burn injury and 20,559 uninjured controls.

There were 232 and 1109 incident ischaemic heart disease admissions in the burn and uninjured cohorts, respectively. After adjusting for confounders, there was a significantly increased rate of first time ischaemic heart disease admissions over the study period (HR, 95% CI: 1.35, 1.16–1.56), with 26% (n = 60) (AR%) of incident ischaemic heart disease admissions attributable to burn injury. Both males (HR, 95% CI: 1.27, 1.08–1.49) and females (HR, 95% CI: 2.15, 1.40–3.31) with burn injury showed increased rates of incident admissions for ischaemic heart disease, when compared with their respective genders in the uninjured cohort.

There were 53 and 228 incident admissions for cerebrovascular disease in the burn anduninjured cohorts, respectively. An increased adjusted rate in first cerebrovascular admissions was found throughout the study period, (HR, 95% CI: 1.46, 1.06–1.99), equating to 32% (n = 16) of incident cerebrovascular admissions attributable to burn injury. Males with burns (HR, 95% CI: 1.43, 1.01–2.05) showed an increased rate of cerebrovascular disease admissions, when compared with uninjured males; however, the elevated rate for females with burns (HR, 95% CI: 1.53, 0.76–3.10) did not achieve significance.

There were 35 and 97 first admissions for heart failure, respectively, in the burn and uninjured cohorts. An increased post-burn rate in first heart failure admissions was found (HR, 95% CI: 1.53, 1.00–2.34), equating to 35% (n = 12) of incident heart failure admissions attributable to burn injury.

## Discussion

It has long been known that severe burns cause long-term systemic effects^1^,^2^ but the potential for non-severe burns to have long-term consequences has, until recently, been largely overlooked. However, non-severe burn injuries account for over 80% of presentations to hospital in developed countries^10^,^11^,^12^ and therefore the potential consequences for patient health and healthcare costs of long-term morbidity associated with non-severe burn are significant.

Here we have shown significant cardiac changes in a clinical study of burn patients with non-severe burn injury. Population level hospital morbidity data comparing burn-injured with non-injured cohorts also suggests long-term effects of burn injury on the cardiovascular system.

The increase in posterior wall thickness and intraventricular septal thickness observed in the mouse model are consistent with the development of hypertrophy associated with sustained sympathetic activation. No statistically significant difference in total body weight or activity level was observed between animals in the control, burn injured or treatment groups, suggesting the cardiac changes are not likely to be solely the result of a sustained hypermetabolic response. The administration of propranolol appeared to ameliorate the changes observed in the mouse model, suggesting that sustained sympathetic activation may underlie the changes observed. However, there are likely to be other factors that can contribute to cardiac changes after a burn injury. Systemic inflammation could cause direct damage to myocytes^13^. Endothelial dysfunction, also related to the inflammatory response, has also been observed after trauma and could contribute to long-term cardiovascular dysfunction^14^. In addition, pain and stress as a consequence of the burn are also likely to contribute to patient outcomes and can significantly impact levels of sympathetic activation and inflammation. These cannot all be accurately reproduced in the murine model, in particular the impacts of pain and stress, since the murine model involves burn injury administered under anaesthesia with immediate analgesia and the use of a full-thickness injury to ameliorate pain and distress.

In the clinical cardiac study, 19 of 24 patients showed an increase in left ventricular end systolic diameter 3 months post burn injury. Radiographically this increase was small but was statistically significant (p = 0.015). In contrast to the animal model, hypertrophic changes in the left ventricular walls were not observed. Patients were not fluid resuscitated at the time of burn injury, heart rate was not significantly different at baseline compared to 3 month post burn injury, and patient BMI remained stable. We propose therefore that neither volume status nor hypermetabolism are causative factors, rather the inflammatory response and/or other factors can be pathological even in a group of patients with an average TBSA of 4.5%. It may be that the extensive and intricate links between sympathetic nervous system (SNS) signals and inflammation^15^,^16^ provide the basis for the efficacy of propranolol administration observed in the murine model, rather than direct effects of β-blockade on cardiac activity after burn injury. However, given the extensive overlap between the stress response, SNS activity and inflammation much more work will be required to fully understand the molecular basis for the cardiac changes observed after non-severe burn injury.

In isolation, cardiovascular specialists would not be concerned by a small increase in LVESD on echocardiography 3 months apart in asymptomatic burns patients; however, coupled with the murine burn model and large population data showing long term cardiovascular morbidity there is a strong case for further clinical investigation. It is also important to consider that the patients included in the clinical study were at increased risk of cardiovascular disease, with 18/24 (75%) of enrolled patients with a BMI of >25 (overweight), a well-known risk factor for cardiovascular disease^17^. This reflects the general trend in Australia for an increasing percentage of adults being overweight or obese, with recent data reporting over 62% of adult Australians have a BMI >25^18^. It is also interesting to note that whilst the average change in LVESD was small, a few patients had larger observed changes in this short time-frame. This may be indicative that there is a subset of patients that are at risk of developing secondary cardiac pathology. This is supported by the population data findings, which whilst showing elevated risk in the burn cohort also showed that only a small proportion of burn patients developed cardiac pathology requiring hospitalisation in the timeframe monitored. Therefore it is likely that the burn injury initiates changes that promote cardiac morbidity that are manifested in a small patient subset. However, the effect could be more significant than observed here as this study only used hospital based data. Assessment of the use of primary care and prescription drugs for cardiovascular disease would provide greater insight into the extent of changes caused by the burns. Given that only a minority of patients may be affected, understanding the mechanisms underlying the pathology could provide an opportunity to identify an ‘at-risk’ patient and therefore to intervene more effectively in patient care. It may also be that burn injury could be classed as a ‘risk-factor’ for CVD, and therefore in those people presenting with other risk factors intervention may be appropriate.

With respect to the population-based study, evaluation of hospital service use for diseases of the circulatory system showed higher rates of admissions for ischaemic heart disease, heart failure and cerebrovascular disease for burn patients when compared to the uninjured cohort. The prolonged time spent in hospital for these cardiovascular conditions, suggests that for those in the burn cohort, the conditions may have been more serious and/or more difficult to manage clinically when compared with the uninjured cohort.

The survival analyses examined admission rates and time to first post-burn circulatory disease admission, and excluded members of the burn cohort who had a record of another non-burn injury admission and potentially additive non-burn injury related systemic effects. After adjustment of confounders, the burn cohort was found to have significantly higher rates of first time or incident post-burn admissions for ischaemic heart disease, heart failure and cerebrovascular disease, with 26%, 35% and 32% of the respective incident admissions being attributed to burn injury, similar to that observed in a study of cardiovascular disease after burn injury in an older population^19^. The population-based study uses hospital service use as a measure of morbidity, and while increased admission rates may reflect new diagnoses, it may also reflect exacerbation of pre-existing disease by burn injury.

Incomplete TBSA% ICD coding may have limited the full understanding of burn severity on long-term hospital use. The study represents both burn injury and cardiovascular diseases serious enough to necessitate hospitalisation and the results may underestimate the extent of the impact of burn injury on cardiac health in the community. Regular quality and accuracy assessments of the Western Australia hospital morbidity data strengthen our findings and it is expected that our results are generalisable to other populations with similar demographic characteristics and comparable health care systems.

These data strongly suggest that non-severe burn injury leads to changes in cardiac function in a subset of patients, rendering them susceptible to secondary pathologies. However, further work to identify the mechanisms leading to these changes is essential to understand the pathophysiology of non-severe burns. In particular inflammation, stress, autonomic activation and endothelial dysfunction all may play a part in the observed cardiac morbidity. Therapeutic intervention will require a better understanding of the relative importance of these changes as a cause of secondary pathology. This may ultimately lead to a method to identify at-risk patients is required to translate these findings into better clinical practice and outcomes for burn patients.

## Methods

### Animal study

Adolescent female C57BL/6J (Animal Resource Centre, Western Australia) mice were maintained in standard housing with food and water provided *ad libitum*. All experiments were approved by The Animal Ethics Committee (AEC) of The University of Western Australia (UWA), and performed in accordance with the National Health and Medical Research Council (NHMRC) Australian Code of Practice for the Care and Use of Animals for Scientific Purposes (AEC ethics number: RA/3/100/1032).

### Full-thickness burn procedure

Full-thickness burn wounds were generated following a validated and previously reported protocol^20^. Female mice were used as they can be accommodated post-injury in group housing without interference in healing of wounds of companion mice. Briefly, mice were anaesthetised in a closed chamber with a continuous flow of 4% isoflurane and high flow oxygen (n = 8 per group, burn injury alone and burn injury + propranolol treatment post-injury). The dorsal area was shaved and the mid lower dorsum received a full-thickness 20 mm diameter burn (8% TBSA) by contact with a brass rod heated to 95 °C and applied to the skin for 9 seconds as previously described. Animals were administered analgesic (buprenorphine, 0.1 mg/kg) subcutaneously in the shoulder immediately post-burn and 12 hours post burn. Continuous analgesia was maintained by administering oral paracetamol (0.01 mg/ml) in drinking water for five days following the procedure. Animals were closely monitored twice daily. Propranolol was administered in drinking water at a concentration of 0.5 mg/ml. Volume of water intake was monitored daily to Mice were approximately 20–25 g and consumed between 3–4 ml water per day equating to approximate intake of 50–100 mg/kg/day.

Echocardiographic studies to measure left ventricular function were performed by Livia Hool (LH) and Helena Viola (HV) as the University of Western Australia

Echocardiography studies of left ventricular function were performed on mice under light methoxyflurane anaesthesia with the use of an i13L probe on a Vivid 7 Dimension (GE Healthcare) with rodent software. Echocardiography was performed on Day 0 (before the burn injury), Day 7, Day 28 and Day 84 post-burn injury. Averages of 3 measurements were taken on M-mode from each mouse at each time-point. M-mode recordings were made at a sweep speed of 200 mm/s. Measurements of left ventricular posterior wall in diastole (LVDPW), left ventricular posterior wall in systole (LVSPW), intra-ventricular septum in diastole (IVDS), and intra-ventricular septum in systole (IVSS), left ventricular end diastolic diameter (LVEDD), left ventricular end systolic diameter (LVESD), fractional shortening (FS), and ejection fraction (EF) were made. Fractional shortening (FS) was calculated by the formula [(LVEDD − LVESD)/EDD] × 100.

### Observational patient study to assess cardiac parameters over time post-burn

This study was approved by the Human Research Ethics Committee, Royal Perth Hospital, Western Australia (EC 2012/038). The study complies in all respects with the National Statement on Ethical Conduct Research Involving Humans and all other national and international ethical requirements. Written informed consent was obtained from all participants.

Inclusion criteria were adult patients admitted to the state burns unit with contact, scald or flame burns. Any part of the body could be burnt and depth of burn could be partial, full-thickness or mixed. Exclusion criteria were; severe burns of ≥15% Total body surface area (TBSA) and/or ≥8% TBSA full thickness; electrical or chemical burns; burns to the chest (that would prevent successful echocardiography); patients with inhalational injuries; past medical history of cardiovascular conditions; currently on prescribed cardiovascular or thyroid medications or steroids; abnormal baseline echocardiogram.

This was a non-randomised, prospective observational study to identify whether there were changes in cardiac parameters in burn patients with non-severe injuries treated with the current standard treatment (not including propranolol). Suitable consenting adult patients underwent baseline Echocardiography (Echo) of the heart within 48 hours of non-severe burn injury. A follow-up Echo was performed at 3 months post injury. 30 patients were recruited and 24 completed both the baseline and 3-month measurements required for the study.

For each case and time-point, a minimum of 3 M-mode or 2D Echocardiograms were taken by a senior sonographer. Patients with abnormal Echocardiography at baseline were excluded from the study. Measurement of cardiac parameters was performed by one cardiology research fellow who was blinded to the identity and time-point of the Echo images.

Cardiac parameters measured included; left ventricular posterior wall (LVPW), intra-ventricular septum (IVS), left ventricular end diastolic diameter (LVEDD), left ventricular end systolic diameter (LVESD), fractional shortening (FS), ejection fraction (EF) and heart rate (HR).

### Statistical analysis

Statistical analysis of animal and patient studies was conducted using Graphpad Prism. For mouse data, all time-points were compared using one-way ANOVA. Patient data were compared using Wilcoxon matched pairs signed rank test. P value of <0.05 was considered statistically significant.

### Population based study

Data from the Western Australian Population-based Burn Injury Project (WAPBIP) were used. The WAPBIP is a population-based retrospective cohort study using linked health administrative data provided from the Western Australian Data Linkage System (WADLS)^21^. The WADLS is a validated record linkage system that routinely links administrative health data from core datasets (including the Hospital Morbidity Data System (HMDS) and the Western Australia Death Register) for the entire population of Western Australia^19^. Quality audits of linkages and data accuracy are undertaken routinely^22^,^23^. The project was approved by the Human Research Ethics Committees of the University of Western Australia and the Western Australian Department of Health (EC 2012/27).

This study used a de-identified extraction of all linked hospital morbidity (HMDS) records for persons 15 to 45 years of age admitted to hospital with a first (index) burn injury in Western Australia, for the period 1 January 1980 to 30 June 2012. The index burn injury was defined as the first hospital admission with a burn injury as the principal and/or additional diagnosis using the International Classification of Diseases (ICD) codes Version 9 (ICD9-CM) 940–949 and Version 10 (ICD10-AM) T20–T31. A population-based comparison cohort was randomly selected from the Western Australian Electoral Roll and Birth Registrations and excluded any person with an injury hospitalisation during the study period. The uninjured comparison cohort was frequency-matched on birth year (4:1) and gender of the burn injury cases for each year from 1980–2012. The project methods have been previously published^24^.

Hospital and death data were linked to each cohort (burn, non-injury) for the period 1980–2012. Hospital admissions data included principal and additional diagnoses, external cause of injury, age and gender, Aboriginal status, index admission and separation dates, burn injury characteristics (total body surface area percent - TBSA%, burn depth, site) and residential postcode. Indices of social disadvantage (Socio-economic Indices for Areas -SEIFA^25^) and remoteness index (Accessibility Remoteness Index of Australia -ARIA+^26^) were supplied for the burn and uninjured cohorts. Mortality data included the date and cause of death. Admissions for diseases of the circulatory system (ICD9 390–459; ICD10 I00-I99, G45) were identified and classified using principal diagnosis data.

Age was categorised into three age groups (15–24; 25–34; 35–44 years). TBSA% was classified into three groups: minor burns (TBSA < 20%), severe burns (TBSA ≥ 20%), and burns for which TBSA% was unspecified. Baseline comorbidity was assessed using the Charlson Comorbidity Index (CCI)^27^ using principal and additional diagnosis fields in the hospital data with a 5-year look back period^28^ (0 CCI = 0; 1 CCI > 0). The social disadvantage index, derived from over 40 census variables and classified as quintiles (from least to most disadvantaged), was included in the models as a proxy measure of lifestyle risk factors (including alcohol use, smoking, physical activity and nutrition)^29^,^30^. The geographic remoteness index was classified into five remoteness categories: major cities, inner regional, outer regional, remote and very remote.

The total number of years a person was at risk (person-years) was estimated from the final discharge date for the burn cases. This date was used for the respective frequency matched non-injury controls. Categorical and non-parametric continuous variables were compared using χ^2^ and Kruskal–Wallis tests, respectively. A P-value of 0.05 or lower was considered statistically significant.

The total number of circulatory disease admissions after burn injury discharge and the summed length of stay for circulatory disease diagnoses were used as outcome measures. The the index burn injury admission was not included in these outcomes. Crude yearly admission rates were calculated and adjusted incidence rate ratios (IRR) with 95% confidence intervals (CI) were generated using negative binomial regression, using all members of the burn injury and uninjured cohorts. Demographic (gender, Aboriginality, age group, social disadvantage, remoteness of place of residence), year of admission (quartiles) and health status variables (any comorbidity at baseline, previous history of circulatory disease admission) were included as covariates in the models.

Cox proportional hazards regression models were performed to assess admission rates for first time hospital use for diseases of the circulatory system post burn discharge. Analyses were conducted on data that excluded records of those with a prior hospitalisation for a circulatory system disease (using a 5-year look back period), with an additional exclusion of those in the burn cohort with other non-burn injury admissions; models were adjusted for demographic and health covariates. The proportional hazard assumption for the burn injured versus non-injured was tested using standard diagnostic tests^31^,^32^.

Attributable risk percentages (AR%) were calculated as the adjusted rate ratio (HR) minus one, divided by the adjusted rate ratio (HR), multiplied by 100^33^. AR% was used to estimate the proportion of incident hospital use for diseases of the circulatory system, where burn injury was a component cause. Statistical analyses were performed using Stata version 12 (StataCorp. LP, College Station, United States of America).

## Additional Information

**How to cite this article**: O’Halloran, E. *et al*. The impact of non-severe burn injury on cardiac function and long-term cardiovascular pathology. *Sci. Rep*. **6**, 34650; doi: 10.1038/srep34650 (2016).

## Figures and Tables

**Table 1:** Summary of demographic details of 24 patients that completed both baseline and 3-month post-injury echocardiography including gender and TBSA of injury.

Gender	TBSA	Age	BMI
F	1.0	33	32
F	2.0	27	24.7
M	2.5	70	34.7
M	3.5	45	34
M	5.0	24	32.9
F	6.0	50	30.6
M	9.0	46	33.8
M	9.0	49	29.8
M	12.0	22	26.8
M	15.0	27	29
M	12.0	19	28
M	5.0	31	32.6
M	3.5	29	34
M	4.0	25	27
M	1.5	22	20.8
M	2.0	27	29
M	3.0	22	23
M	7.0	19	23
M	12.0	44	32
M	3.0	27	33.7
M	3.0	20	21
M	10.5	55	30
M	1.5	19	24.8
M	10.0	44	29.6

**Table 2:** Baseline demographic and pre-existing health status factors for those aged 15 to 45 years at first burn injury hospitalisation and frequency matched non-injury cohort, Western Australia, 1980–2012.

Characteristics	No Injury N (%)	Burn injury N (%)	p-value
*Total*	56,822	14,555	
*Demographic*			
Aboriginal			
Yes	767 (1.3)	1,764 (12.1)	<0.001
Social disadvantage quintiles*			
Quintile 1. (Most disadvantaged)	5,977 (10.5)	2,918 (20.5)	<0.001
Quintile 2.	12,729 (22.5)	4,683 (32.9)	
Quintile 3.	10,473 (18.5)	3,089 (21.7)	
Quintile 4.	10,454 (18.4)	1,793 (12.6)	
Quintile 5. (Least disadvantaged)	17,030 (30.1)	1,748 (12.3)	
Remoteness**			
Major city	42,989 (75.9)	7,031 (49.2)	<0.001
Inner regional	4,700 (8.3)	1,577 (11.0)	
Outer regional	4,867 (8.6)	2,407 (16.9)	
Remote	2,491 (4.4)	1,732 (12.1)	
Very remote	1,614 (2.8)	1,532(10.7)	
*Health status*			
Any comorbidity (CCI>=1)†	729 (1.3)	803 (5.5)	<0.001
Prior admission for disease of	829 (1.5)	564 (3.9)	<0.001
circulatory system‡			

^*^SEIFA socio-economic disadvantage quintiles; 2.2% missing values for burn and 0.3% for uninjured.

^**^ARIA+ remoteness classification; 1.9% missing values for burn and 0.3% for uninjured.

^†^Comorbidity based on derived Charlson Comorbidity Index (CCI) using 5-year look-back.

^‡^Principal diagnosis record of hospitalisation for circulatory disease (ICD9 390–459; ICD10 I00-I99, G45) using 5-year look-back period.

**Table 3:** Classification (percentage) of post-burn discharge admissions for primary diagnosis diseases of the circulatory system for those aged 15 to 45 years hospitalised for burn injury.

Classification of admissions for diseases of circulatory system	Percentage (%)
Acute rheumatic heart diseases	0.2
Chronic rheumatic heart diseases	0.7
Hypertensive diseases	3.1
Ischaemic heart diseases	32.4
Pulmonary heart disease & diseases of pulmonary circulation system	1.5
Heart failure	8.6
Other forms of heart disease	13.9
Cerebrovascular diseases	5.7
Diseases of arteries, arterioles and capillaries	5.6
Diseases veins, lymphatic vessels, lymph nodes not elsewhere classified	26.5
Other and unspecified disorders of the circulatory system	1.8
Total	100%
